# Bacteriophage application restores ethanol fermentation characteristics disrupted by *Lactobacillusfermentum*

**DOI:** 10.1186/s13068-015-0325-9

**Published:** 2015-09-04

**Authors:** Mei Liu, Kenneth M. Bischoff, Jason J. Gill, Miranda D. Mire-Criscione, Joel D. Berry, Ry Young, Elizabeth J. Summer

**Affiliations:** Ecolyse Inc., 11142 Hopes Creek Rd., College Station, TX 77845 USA; Renewable Product Technology Research Unit, National Center for Agricultural Utilization Research, Agricultural Research Service, U.S. Department of Agriculture, 1815 N. University St., Peoria, IL 61604 USA; Center for Phage Technology, 2128 TAMU, Texas A&M University, College Station, TX 77843 USA; Department of Animal Science, 2471 TAMU, Texas A&M University, College Station, TX 77843 USA; Department of Biochemistry and Biophysics, 2128 TAMU, Texas A&M University, College Station, TX 77843 USA

**Keywords:** Lactic acid bacteria, *Lactobacillus fermentum*, Ethanol fermentation, Bacteriophage

## Abstract

**Background:**

Contamination of corn mash by lactic acid bacteria (LAB) reduces the efficiency of the ethanol fermentation process. The industry relies heavily on antibiotics for contamination control and there is a need to develop alternative methods. The goals of this study were to determine the diversity and abundance of bacteria contaminating commercial ethanol fermentations, and to evaluate the potential of anti-LAB bacteriophages in controlling production losses.

**Results:**

Bacterial populations in 27 corn mash samples collected from nine different commercial plants were determined by pyrosequencing of 16S rRNA amplicons. The results showed that the most abundant bacteria (>50 % of total population) in 24 of the 27 samples included LAB genera such as *Lactobacillus*, *Streptococcus*, *Lactococcus*, *Weissella*, *Enterococcus*, and *Pediococcus. Lactobacillus* was identified as the most prevalent genus at all fermentation stages in all plants, accounting for between 2.3 and 93.7 % of each population and constituting the major genus (>50 %) in nine samples from five plants and the most abundant genus in five other samples. *Lactobacillus* species, including *L. delbrueckii*, *L. fermentum*, *L. mucosae*, and *L. reuteri* were the most well-represented species. Two bacteriophages that target *L. fermentum* strains from ethanol plants, vB_LfeS_EcoSau and vB_LfeM_EcoInf (EcoSau and EcoInf), were isolated and characterized as a siphophage and a myophage, respectively. Analysis of the 31,703 bp genome of EcoSau revealed its similarity to the P335-like phage group, and the 106,701 bp genome of phage EcoInf was determined to be a novel phage type despite its distant relationship to the SPO1-like phages. Addition of phages EcoSau and EcoInf to *L. fermentum*-contaminated corn mash fermentation models restored the yields of ethanol and reduced levels of residual glucose, lactic acid, and acetic acid to that comparable to the infection-free control.

**Conclusions:**

This study provides detailed insight into the microbiota contaminating commercial ethanol fermentations, and highlights the abundance of LAB, especially *L. delbrueckii*, *L. fermentum*, *L. mucosae*, and *L. reuteri*, in the process. This study suggests that phages with broad coverage of major LAB species can be applied directly to corn mash for antibiotic-free control of contamination in the ethanol fermentation industry.

**Electronic supplementary material:**

The online version of this article (doi:10.1186/s13068-015-0325-9) contains supplementary material, which is available to authorized users.

## Background

Commercial biofuel ethanol fermentation plants utilize starch or sugar as substrate and rely on yeast for fermentation. Biofuel ethanol fermentation is not a sterile process: during normal operation, 10^5^–10^8^ colony forming units (cfu) per milliliter of bacteria may be present in the system [1]. Lactic acid bacteria (LAB) are problematic for the industry not only through competition for the fermentation feedstock but also through the generation of lactic and acetic acid byproducts that inhibit yeast growth [2–6]. Even a single log^10^ reduction in the amount of LAB can increase ethanol yield by approximately 3.7 % [5]. Common approaches used by fermentation plants to reduce LAB contamination include sanitization and the addition of antibiotics such as virginiamycin and penicillin [7]. However, none of the currently used approaches are perfect and fermentation plants continue to experience LAB-associated yield losses [1, 7]. There is also increasing pressure to reduce the potential for antibiotic residues in the dried distillers’ grains that are sold post-process for animal feeds [5, 8]. Therefore, there is a need to develop new control methods that are not based on chemical antibiotics. One such approach, evaluated here, is the use of bacteriophages.

The diversity of the contaminating LAB in biofuel ethanol fermentation has been analyzed extensively using culture-based approaches. The predominant group of bacteria isolated from corn-based ethanol fermentation facilities are reported to be LAB genera *Lactobacillus*, *Bifidobacterium*, *Lactococcus*, *Leuconostoc*, *Pediococcus*, and *Weisella* [1, 9]. The LAB species most frequently isolated from fermentation vessels are *Lactobacillus* species, including *L. fermentum*, *L. vini*, *L. johnsonii*, *L. mucosae* and *L. amylovorus* [1, 7, 8]. However, culture-based approaches are subject to well-known limitations and broad, culture-independent surveys of bacterial diversity at commercial corn ethanol fermentation facilities are not available. Similarly, LAB phages are among the most extensively catalogued of all phages, primarily due to their well-documented detrimental impact on food fermentation processes [10–12]. Over 1000 phages of *Lactococcus* and *Streptococcus* alone have been described at least to the level of virus particle morphology [13]. Phages infecting dairy strains of *Lactococcus lactis* and *Streptococcusthermophilus* have been the focus of considerable analysis, with 66 and 11 complete genome sequences available, respectively [11, 14]. However, phages capable of broadly controlling the LAB populations present specifically in ethanol fermentation facilities have not been described.

Phage-based antibacterial agents have been evaluated in various medical, agricultural, and industrial settings [6, 15–18]. The use of phage is complicated by the tendency for most phage to infect an extremely limited number of host strains, frequently only one or a few strains of a given species. Therefore, developing a phage-based product necessitates a detailed understanding of the real diversity present in the targeted system in order to ensure the inclusion of phages with sufficient host coverage. Additionally, phage efficacy testing needs to include all components present in the industrial process. Fully developing a phage preparation that can be used in commercial fermentation plants requires isolation of phages that show killing activity against the same diversity of LAB strains present in commercial plants, as well as demonstration of phage killing activity in a corn mash matrix. Previous studies demonstrated that phages were capable of controlling *L. plantarum* ATCC 8014 when co-cultured with yeast in defined liquid culture media [19]. Purified phage lytic enzymes were shown to be able to lyse *Lactobacillus* strains in both culture media and in mock fermentations using corn fiber hydrolysates as substrates [20]. In addition, expression of phage lytic enzymes in yeast reduced *L. fermentum*, as well as lactic acid and acetic acid levels during corn mash fermentations [21]. In the research presented here, bacterial diversity was analyzed in commercial corn mash samples and phages capable of killing the predominant LAB strains were isolated and tested for their effects on the end fermentation products in a corn mash fermentation assay.

## Results

### Bacterial population analysis in commercial corn mash during early, middle, late stage fermentation

Between July 2011 and August 2012, samples were collected from nine different commercial corn ethanol fermentation plants (Table 1). Samples were collected from fermentors during early, middle, and late fermentation stages, all pH ~4–4.5 and containing yeast at 8 × 10^7^–2 × 10^8^ cfu/g. Culturable bacterial counts were found to vary greatly, ranging from 10^3^ to 10^7^ cfu/g. There was no direct correlation between the total bacterial counts and the stage of the fermentation (Table 1).

**Table 1:** Bacterial population diversity at early, mid, and late fermentation stages in nine different commercial ethanol plants

Plant	Fermentation stage (hours)	Bacteria level (CFU/g)	# of total OTU	# of LAB OTU	*Lactobacillus* (%)	*Streptococcus* (%)	*Lactococcus* (%)	*Weissella* (%)	*Enterococcus* (%)	*Pediococcus* (%)	Total LAB (%)^a^
1	11	NA	68	7	36.10						36.10
	24	NA	98	12	91.91	0.02			0.01		91.94
	52	NA	21	6	91.09	0.78					91.87
2	25	4.4E+07	16	10	5.78	0.51	0.65	92.22	0.07		99.23
	34	1.9E+07	15	10	3.62	0.61	0.47	79.50		0.10	84.30
	43	9.4E+06	15	12	16.09	9.98	3.42	70.10			99.59
3	15	8.6E+04	30	21	4.53	0.18	63.08	30.34	0.02		98.15
	30	3.6E+04	19	10	47.36	0.35	0.35	39.58			87.64
	45	1.7E+04	24	9	16.54		0.44	75.51			92.49
4	9	1.6E+07	55	15	7.32	4.93	0.66	11.86			24.77
	27	1.8E+06	19	13	54.70	25.07	0.33	18.47			98.57
	45	7.8E+04	17	11	82.66	8.92	1.48	5.85			98.91
5	18	1.2E+07	11	8	40.64		58.42	0.64	0.07		99.77
	32	1.8E+06	18	9	75.00		23.51	0.06			98.57
	45	8.6E+05	14	5	70.55		2.47		25.60		98.62
6	8	5.6E+05	18	17	28.56	69.03			0.75		98.34
	26	2.5E+05	26	18	81.77	8.84			5.11	0.41	96.13
	34	1.0E+07	12	11	93.71	6.18					99.89
7	15	4.4E+03	25	7	13.15	4.21	46.31		0.52	3.16	67.35
	34	2.8E+05	41	9	39.46	5.54	5.54				50.54
	42	2.8E+04	18	6	17.61	1.14	68.47				87.22
8	17	2.2E+06	7	7	2.31	97.54					99.85
	35	7.8E+05	8	5	67.46	22.81			9.20		99.47
	54	7.0E+03	14	9	38.86	23.45			36.40		98.71
9	6	3.0E+05	23	8	14.20			0.71		41.84	56.75
	24	2.5E+05	19	7	26.94	0.52				54.92	82.38
	49	3.0E+04	32	6	29.34					14.97	44.31

Values less than 0.01 % or not detected are not shown

*OTU* operational taxonomic units, *N*A data not available

^a^% of LAB is calculated as the sum at *Lactobacillales* order level

Bacterial population analysis was conducted by 16S amplicon pyrosequencing. Between 2124 and 27,188 (average of 5025) 16S sequences were generated from each sample (Table 1). A significant proportion of these were identified as probably plastid in origin and excluded from subsequent population analysis. The sequences were clustered into operational taxonomic units (OTU) at 1 % divergence, between 7 and 98 per sample, for a total of 243 OTU in all 27 samples (Table 1). Of the 243 OTU, 51 belonged to members of the LAB. LAB-derived sequences were overall the most common, from 36.1 to 99.9 %, in every sample, and were the most abundant (>50 % of total population) in 24 of the 27 samples (Table 1). LAB genera present in the samples included *Lactobacillus*, *Streptococcus*, *Lactococcus*, *Weissella*, *Enterococcus* and *Pediococcus.* Of these, *Lactobacillus* was the most widespread genus, with representatives identified in all 27 samples from all nine fermentation facilities at 2.3–93.7 % of the population and constituting the major genus (>50 %) in nine samples from five plants and the most abundant genus in five other samples. *Streptococcus*, *Lactococcus* and *Weisella* species were also well represented, being found in 20, 15 and 12 samples, respectively, but were the majority in fewer samples (two, four and five, respectively). The less widely distributed genera were not necessarily the least abundant genera. For example, while *Pediococcus* species were present in only six samples, *Pediococcus* was the most abundant bacterial genus in one plant (Plant 9), accounting for 41.8, 54.9, and 15.0 % of the total bacteria in early, mid, late stage fermentation, respectively (Table 1).

**Fig. 1:**
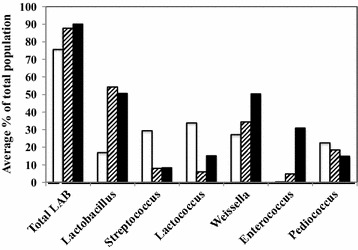
Average percentages of total LAB and different LAB genera at different fermentation stages. Early (*empty bar*), mid (*striped bar*), and late (*filled bar*) fermentation samples are presented. Values were calculated using only samples in which those populations were present

The average abundance of each bacterial genus, calculated using only the samples in which each genus was present, varied depending on the stage of fermentation (Fig. 1). While overall LAB levels increased from early to late stage fermentation, from 75.6 to 90.2 %, the increase was not observed across all genera. *Lactobacillus*, *Weissella*, and *Enterococcus* levels were significantly higher at late stage as compared to early stage fermentation. In contrast, *Streptococcus*, *Lactococcus* and *Pediococcus* levels were reduced in late stage fermentation samples as compared to the early stage fermentation samples (Fig. 1). It should be noted that not every sample contained every genus so the data set is more robust for *Lactobacillus* and *Streptococcus* than for organisms present in fewer samples. When the sample distribution of different species was analyzed, several particularly predominant LAB species (defined as being present at ≥20 % of total population in any sample tested) were identified (Table 2). These notable species included *Enterococcus faecium*, *Lactobacillus delbrueckii*, *L. fermentum*, *L. mucosae*, *L. reuteri*, *Lactococcus lactis*, *Pediococcus pentosaceus*, and *Weissella confusa*, and their relative abundance varied from sample to sample (Table 2).

**Table 2:** Predominant bacterial species of nine different commercial ethanol plants

Plant	Fermentation stage (hours)	*Enterococcus faecium* (%)	*Lactobacillus delbrueckii* (%)	*Lactobacillus fermentum* (%)	*Lactobacillus mucosae* (%)	*Lactobacillus reuteri* (%)	*Lactococcus lactis* (%)	*Pediococcus pentosaceus* (%)	*Weissella confusa* (%)
1	11			0.3	9.5	6.1			
	24		0.8	1.2	*42.5*	*29.5*			
	52			*42.4*		*32.6*			
2	25			4.7	0.4				*48.2*
	34			3.2	0.3			0.1	*46.9*
	43			11.8	0.4				*44.7*
3	15		2.3	0.4	0.7		*36.7*		16.3
	30		*31.3*	13.7	0.2		0.4		*21.8*
	45		11.9	1.8					*67.3*
4	9		11.3	5.3	9.6		0.9		*33.2*
	27		*26.5*	0.9	15.1		0.9		14.6
	45		*45.9*	3.2	18.8				5.8
5	18		0.5	0.6	5.0		*34.0*		0.4
	32		4.1	1.4	*67.8*		14.1		0.5
	45		4.9		*65.2*		0.9		
6	8	2.3	0.4	0.2	4.9	1.5			
	26	4.9	0.7	0.6	*35.5*	15.8		0.2	
	34	0.3		0.4	9.9	1.4			
7	15			4.7	0.7	2.3	*28.2*	2.3	
	34		0.2	*29.7*	3.5	0.7	3.4		
	42		2.1	0.7	15.7	0.2	*37.0*		
8	17			1.2	0.3				
	35	8.9		*54.1*	11.3				
	54	*36.4*		*28.7*	6.3				
9	6			1.4	8.5	0.3		*44.3*	
	24		0.3	1.9	9.3			*54.2*	0.3
	49			0.5	15.5	0.2		13.5	

Predominant species are defined as those present at ≥20 % of total bacterial population (italic numbers) in any sample. Values not detected or less than 0.1 % are not shown

The patterns of abundance and distribution of non-LAB OTU were quite different from that of the LAB. While 192 non-LAB OTU were identified, the distribution among the samples was uneven—ranging from just one to 86 different OTU per sample. The average number of non-LAB OTU in each sample was 15 (±19.5) while the average number of LAB OTU in each sample was 10 (±4.0). In order to represent the number of shared species between each sample, the Sørensen–Dice index was calculated for each pairwise comparison (Table 3). This calculation presents the shared OTU as a function of potentially shared OTU, generating a value between 0 (no shared OTU) and 1 (100 % shared OTU, which is the value obtained when a sample is compared to itself). Values closer to 1 indicate a greater proportion of the potentially shared OTU is present in both samples.

**Table 3:**
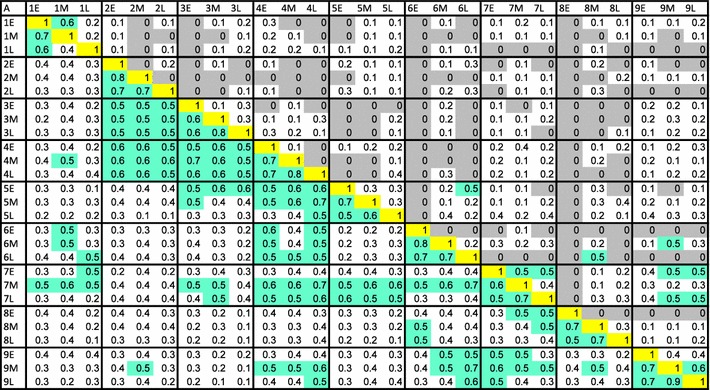
Pair-wise comparison of LAB and non-LAB shared species among early, mid, and late fermentation samples of nine plants

Sørensen–Dice index values were presented and color-coded: gray (no species in common), white (up to 0.4), green (greater than 0.4), yellow (all species shared between the two samples). Values above the yellow diagonal are comparisons of non-LAB, values below yellow diagonal are comparisons of LAB

The pairwise comparisons were generated between every sample for both LAB and non-LAB genera (Table 3). Several patterns emerged from this analysis. First, the LAB populations were more uniform across samples than the non-LAB populations. The average index calculated for LAB was 0.401 ± 0.147 and for non-LAB was 0.113 ± 0.129. These values indicate that despite the fact that many more types of non-LAB bacteria were identified in the 27 samples, each type was present in fewer samples. Many of the non-LAB pairwise comparisons were zero, indicating that the non-LAB component of most samples was completely unlike that of other sample. In contrast, distribution of LAB was more uniform as all samples contained at least one LAB in common with every other sample (Table 3).

In several samples, the non-LAB genera identified included organisms known to be problematic in other industrial settings, due to their corrosion, fouling or sulfidogenic capacity (Table 4). Notable genera included acetogenic bacteria such as *Acetobacterium*, present in seven out of the 27 samples. *Acetobacterium* species are acid producing that ferment alcohols into acetic acid. Iron-reducing bacterial genera, including *Shewanella* and *Geobacter*, were present in ten of the 27 samples. Sulfidogenic bacteria, capable of evolving hydrogen sulfide, were present in 15 of the 27 samples. These sulfidogens included sulfate reducing bacteria (SRB) such as *Desulfovibrio* and *Desulfotomaculum*, as well as non-SRB sulfidogens such as *Citrobacter.* The samples with the greatest diversity of non-LAB populations (Plants 1 and 4, with 61 and 86 non-LAB genera, respectively) contained numerous isolates of sulfidogenic bacteria and iron-reducing bacteria. Interestingly, these organisms were present primarily in the early and mid-stage fermentation samples.

**Table 4:** Non-LAB genera in commercial ethanol plants

Metabolic trait	OTU	Samples	Example genera
Sulfidogen, all	22	15	*Desulfovibrio, Dethiosulfovibrio*
Sulfate reducing bacteria	12	8	*Desulfotomaculum*
Other sulfidogens	10	12	*Citrobacter*
Iron reducing bacteria	7	10	*Shewanella, Geobacter*
Acid producing bacteria, all^a^	59	27	*Alicyclobacillus*
Acetogen	3	7	*Acetobacterium*

*OTU* operational taxonomic units

^a^Acid producing bacteria include lactic acid bacteria

### Isolation of bacterial strains from commercial ethanol plants

Corn mash samples were serial diluted and from the highest dilution plates, bacterial colonies representing dominant colony morphological types were isolated. These dominant isolates, both numerically and morphologically, were identified by sequencing of 16S amplicons. The isolated strains included multiple representatives of 36 species from the LAB genera including *Enterococcus*, *Lactobacillus*, *Lactococcus*, *Leuconostic*, *Pediococcus*, *Streptococcus*, and *Weissella*. The identities of the ethanol plant isolates are consistent with the bacterial diversity survey. These LAB isolates were used as hosts for phage isolation and characterization.

### Isolation and characterization of *L. fermentum* phages EcoSau and EcoInf

Bacteriophages showing killing activity against fermentation plant LAB were isolated. Two of these, phages vB_LfeS_EcoSau and vB_LfeM_EcoInf, were isolated from commercial sauerkraut and municipal wastewater influent water, respectively, using host *L. fermentum* 0315-25. *L. fermentum* 0315-25 was isolated from commercial corn mash and shown to be capable of reducing ethanol yields in an infected fermentation assay [5, 8]. These two phages are hereafter referred to as EcoSau and EcoInf, respectively. The host ranges of EcoSau and EcoInf were assessed against *L. fermentum* and *L. mucosae* isolated from different commercial ethanol plants. EcoInf was active against all 12 *L. fermentum* strains tested (representing eight plants) and one *L. mucosae* strain. EcoSau was active against ten *L. fermentum* strains (representing six plants) and two *L. mucosae* strains from one plant (Table 5).

**Table 5:** Activities of EcoSau and EcoInf against *L. fermentum* and *L. mucosae* isolates

	Strains tested	Plants represented	Susceptible to EcoSau^a^		Susceptible to EcoInf^a^	
			Strains	Plants	Strains	Plants
*L. fermentum*	12	8	10	6	12	8
*L. mucosae*	8	4	2	1	1	1

^a^Susceptibility assayed by spotting 10 µl of a routine test dilution of phage (~105 pfu/ml)

**Fig. 2:**
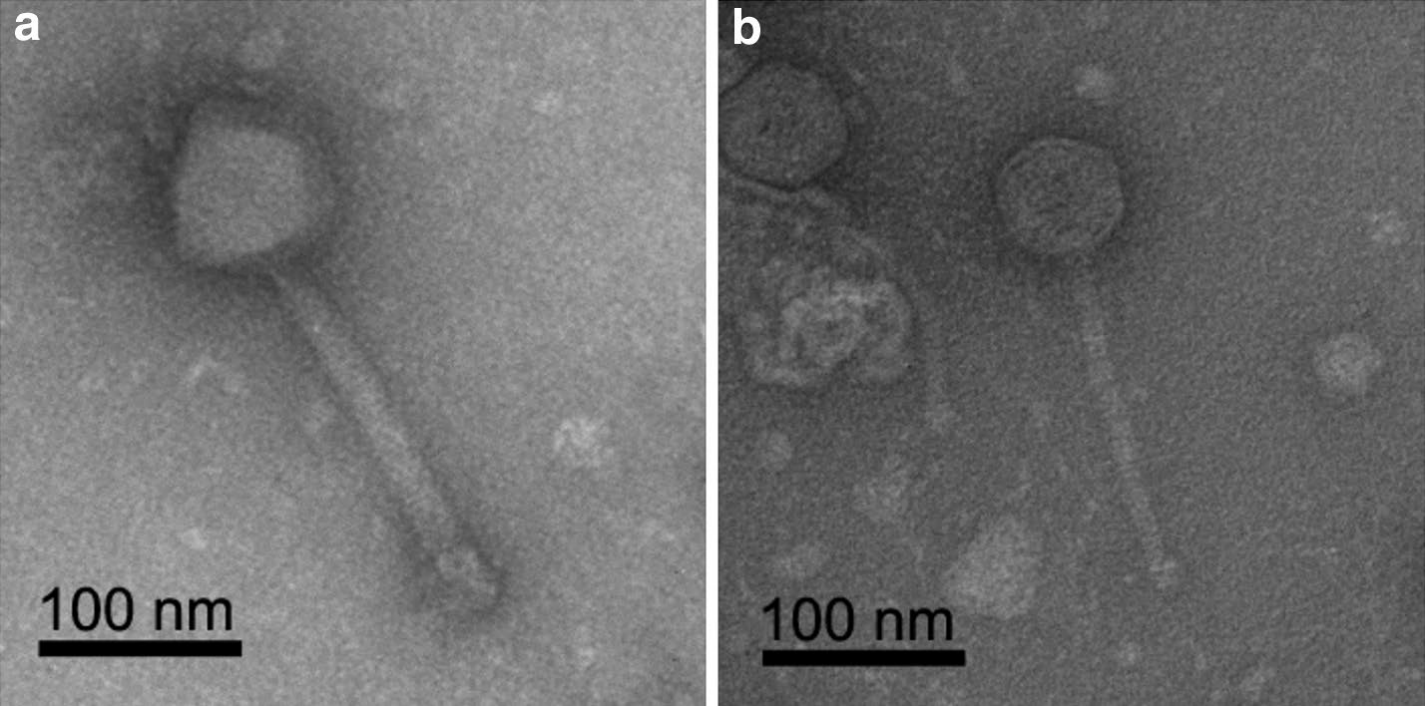
Transmission electron micrographs of phages EcoInf (**a**) and EcoSau (**b**). *Bars* 100 nm

The morphologies of phages EcoSau and EcoInf were determined by TEM (Fig. 2; Table 6). EcoSau virions consisted of long (~179 nm) flexible, non-contractile tails with an average width of ~10 nm, and isometric capsids with an average diameter of ~62 nm, suggesting a *T* = 7 icosahedral symmetry as seen in many siphophages, including the well-studied phages lambda and T1. EcoInf virions exhibited thick (20 nm), non-flexible, contractile tales of ~202 nm and isometric heads with an average diameter of ~89 nm, typical of SPO1-like phages [22]. Morphologically, EcoSau and EcoInf were categorized as siphophage and myophage, respectively.

**Table 6:** Summary of the characteristics of phages EcoSau and EcoInf

Phage	Morphology	Phage group	Head diam (nm)	Tail length (nm)	Tail width (nm)	Genome length (bp)	GC (%)	Genomic termini	No. of CDS	tRNA
EcoSau	*Siphoviridae*	P335-like	62 ± 2	179 ± 6	11 ± 1	31,703	48.4	*pac*	50	No
EcoInf	*Myoviridae*	A novel type^a^	89 ± 3	202 ± 4	20 ± 1	106,071	38.2	Direct terminal repeat (817 bp)	124	Yes (2)

^a^Very distantly related to SPO1-like phages

### Genomic analysis of phages EcoSau and EcoInf

**Fig. 3:**
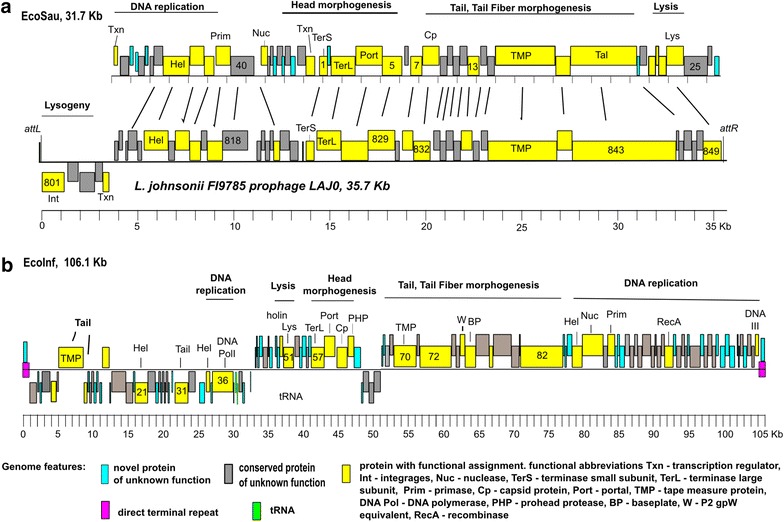
Genome maps of EcoSau and EcoInf. Predicted genes are represented by *boxes above* and *below* the *heavy black line*; *boxes above the lines* are genes encoded on the forward strand, and those below the lines are on the reverse strand. The *ruler below* the genomes indicates the scale (in kb). Genome features (novel or conserved proteins of unknown function, proteins with functional assignment, tRNA genes, terminal repeat) are *color coded* according to the legend. *Lines above* the map show the extent of the suggested functional modules. **a** Comparison map of EcoSau to *L. johnsonni* FI9785 prophage LAJO. The LAJO prophage encompasses the region encoding locus tags FI9785_801 to FI9785_849 (accession no. NC_013504). Putative attL and attR sites on the prophage genomic termini are indicated. Genomic map of EcoSau was opened between gp 27 and gp 28 for easy comparison and alignment with the rest two genomes. Proteins sharing identities (*e* value <10^−5^) were linked with *black lines*. **b** Genome map of EcoInf

Phages EcoInf and EcoSau were further characterized by whole genome sequencing (Table 6). Functional clusters encoding virion morphogenesis, DNA metabolism, and host cell lysis could be identified in both phages (Fig. 3). Annotation tables for phages EcoSau and EcoInf are shown in Additional files 1, 2: Tables S1, S2, respectively. The EcoSau genome was determined to be 31,703 bp and predicted to contain 50 protein-coding genes and no tRNA genes. The EcoSau genome produced a circular assembly, suggesting that the phage utilizes a *pac* type packaging mechanism [23]. The phages most similar to EcoSau, defined as having the most shared proteins, were members of the P335 group of phages, in particular *Lactococcus* phages TP901-1, Tuc2009, and P335 (Additional file 3: Table S3) [24, 25]. The shared genes were located primarily in the virion morphogenesis modules, and included terminase, scaffold, major and minor tail proteins. Conservation between EcoSau and prophage elements located in the genome entries of several LAB strains was also evident. The most similar of these to EcoSau was found to be a 35.7 kb prophage in the *L. johnsonii* FI9785 genome, with 25 EcoSau proteins exhibiting similarity (*e* < 10^−5^) to FI9785 prophage proteins (Fig. 3; Additional file 3: Table S3). It is likely, however, that EcoSau is a virulent phage as the region of the EcoSau that would encode the lysogeny module is deleted. It should be noted that the P335 group of phages includes both temperate and virulent members [26].

The EcoInf genome consisted of 106,071 bp of unique sequence and was predicted to encode 124 proteins and two tRNAs (Fig. 3; Table 6). Functional annotations could not be made for 101 of the 124 EcoInf predicted proteins, as 52 exhibited similarity only to other proteins of unknown function and 49 had no recognizable homologs in the NCBI nr database. Despite the predominance of novel proteins, candidate genes involved in virion morphogenesis, DNA replication, and host cell lysis genes were identified. The lack of conservation between EcoInf and phage elements at a DNA and protein level suggests that EcoInf is a novel phage type. Phages most similar to EcoInf were of the SPO1-like group, with 41 out of 124 predicted EcoInf proteins sharing at least some level of protein sequence similarity (25–62 %), to proteins present in the genomes of SPO1-like phage group members, in particular *Lactobacillus* phage LP65 and *Bacilluspumilus* phage phiAGATE (Additional file 4: Table S4) [25, 27]. To date, the genomes of all large (>90 kb) myophages of Gram-positive bacteria in the Refseq database have characteristics common to a diverse group designated as SPO1-like, with the founding member being a paradigm phage of *B. subtilis*, SPO1 [27, 28]. However, the EcoInf genome is at least 15 kb smaller than any SPO1-like phage. The size difference was in part due to the genomic termini of EcoInf. The SPO1-like phages have direct terminal repeats, of at least 3 kb and usually 8–10 kb, encoding multiple proteins, whereas EcoInf has a terminal repeat of 817 bp, encoding a single protein. EcoInf, similar to SPO1-like phages in general, appears to be a virulent phage as no genes involved in prophage maintenance or lysogeny were identified.

### Phage effect in ethanol fermentation contaminated with *L. fermentum*

**Fig. 4:**
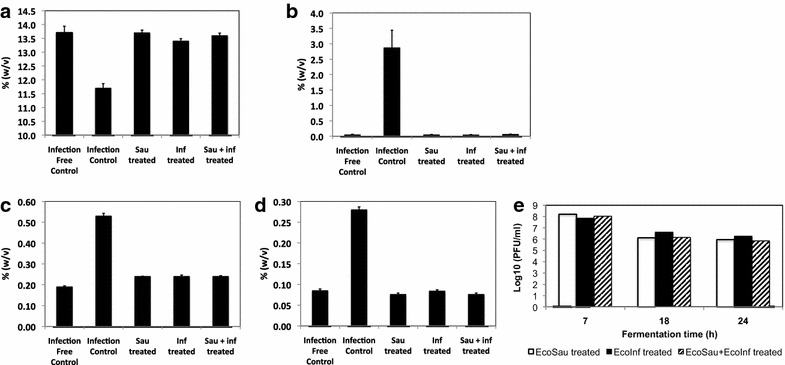
Effect of phages EcoSau and EcoInf in ethanol fermentation models contaminated with *L. fermentum* at 10^7^ cfu/ml starting level. Fermentation groups were set up in triplicate, including infection-free control (no bacteria challenge), infection control (bacteria challenged without treatment), and EcoSau, EcoInf, or EcoSau + EcoInf treatment (bacteria challenged and with phage treatment at MOI of 10). At the end of the fermentation, levels of **a** ethanol **b** glucose **c** lactic acid, and **d** acetic acid, were determined and compared. Levels of recoverable phage (**e**) were determined during fermentation in phage-treated systems

Shaker-flask fermentation models simulating bacterial contamination during corn ethanol fermentation were established. In these tests, 250 ml flask fermentations containing *Saccharomyces cerevisiae* growing on saccharified corn mash were incubated for 72 h, either without added bacteria (infection-free control) or challenged with *L. fermentum* strain 0315-25 at an inoculation level of 10^7^ cfu/ml (infection control). Some of the *L. fermentum*-challenged flasks were also treated with phage EcoInf and EcoSau, either singly or in combination. The effect of phage treatment on *L. fermentum*—contaminated fermentation was determined by measuring levels of residual glucose, lactic acid, acetic acid, as well as final ethanol yields of the fermentation systems. Cultures challenged with bacteria showed decreased ethanol yields, while phage-treated samples showed full recovery of ethanol production. At 72 h, ethanol levels in the bacterial challenged systems without phage treatment (infection control) were 11.7 % (w/v), whereas the phage-treated systems attained yields of ~13.5 % w/v, comparable to the infection-free control cultures (Fig. 4a). Residual glucose is an indicator of fermentation completeness; phage-treated and infection-free cultures contained similar residual glucose levels of ~0.05 %, compared to 2.8 % residual glucose in the infection control (Fig. 4b). Acid accumulation is indicative of infection by lactic acid bacteria. In systems challenged with bacteria but not treated, the levels of lactic and acetic acid were quite high, 0.53 % (w/v) and 0.28 % (w/v), respectively. In contrast, lactic acid levels in the bacteria-free samples and in the phage-treated samples were lower, 0.19 % (w/v) to 0.24 % (w/v) (Fig. 4c). Similarly, acetic acid levels in the bacteria-free and phage-treated samples were approximately 0.08 % w/v (Fig. 4d). These results indicate that all three phage treatments (EcoSau alone, EcoInf alone, and EcoSau and EcoInf combined) could restore the levels of ethanol, glucose, lactic acid, and acetic acid in the infected systems to those observed in the infection-free control. Compared to the inoculated phage levels at the start of the experiments (10^8^–10^9^ pfu/ml), the levels of recoverable phage decreased by approximately 100- to 1000-fold in the phage-treated corn mash systems by 24 h (Fig. 4e), possibly due to phage inactivation or adsorption to corn mash particles.

## Discussion

The economics of the biofuel fermentation process precludes the level of sterility required to prevent bacterial contamination without the use of antibacterial agents. The potential for phage to be used to control LAB during fermentations has been speculated. The capacity for phage to control LAB is aided by the short time frame of fermentation (typically 50–60 h) and the batch nature of the process, both traits that limit the time and volume needed to be treated. However, the majority of phages have very narrow host ranges, limited to one or a few strains for a given species. Therefore, the prophylactic use of phage requires extensive knowledge of the target bacteria both in terms of diversity and abundance. Primarily culture-based enumeration methods have implicated LAB and *Lactobacillus* spp. in particular, as the dominant microbial contaminant during ethanol fermentation [1, 7, 8]. This is not unexpected given that LAB are well documented to dominate fermentation of many plant materials [1, 9]. In this study, both culture-based and 16S bacterial pyrosequencing analysis confirmed the total and relative abundance of LAB, with LAB accounting for greater than 90 % of the total population in the majority of the commercial fermentors tested. *Lactobacillus* species, including *L. delbrueckii*, *L. fermentum*, *L. mucosae*, and *L. reuteri* were the most well-represented species. In addition to *Lactobacillus*, other LAB genera including *Lactococcus*, *Streptococcus*, and *Weissella* were also identified. In this study, a range of 10^3^–10^7^ cfu/ml contaminating bacteria was detected in fermentors of commercial ethanol plants. LAB levels reported here are consistent with previous work that reported LAB levels of 10^5^–10^8^ cfu/ml in fermentors in the US and Brazil [1, 29]. It should be noted that numerical abundance does not de facto implicate the capacity to cause fermentation slowdown. The correlation between the numerical dominance of other species or strains and their impact on fermentation loss is not clearly defined. Many members of these predominant species are capable of using obligately and/or facultatively heterofermentative metabolism and produce a variety of end products including ethanol, lactic acid, acetic acid, CO_2_, and mannitol [30]. The metabolic traits of these lactic acid bacteria might make them more dangerous in inhibiting yeast fermentative activity compared to homofermentative lactic acid bacteria and non-acid producing bacteria, since lactic acid and acetic acid work synergistically in reducing the growth rate of yeast, decreasing its glucose consumption rate and ethanol production [2–5]. However, studies have demonstrated that even closely related LAB isolates exhibit differences in their capacity to inhibit yeast fermentation, indicating that fermentation slowdown is not entirely explained as a secondary effect of acid production [5, 8].

The two phages isolated and evaluated in this study, EcoSau and EcoInf, both exhibited broad host range, infecting the majority of *L. fermentum* stains isolated from multiple commercial plant corn mash samples. Even though some 230 *Lactobacillus* phages have been described, and genomic sequence data is available for over 20 of these, EcoSau and EcoInf were found to represent novel phage types, with only limited similarity to previously characterized phages [31]. EcoSau was found to be most similar to the *Lactococcus* P335-like phages, a group that includes closely related temperate and virulent isolates that interfere with dairy starter cultures [24, 25, 32]. EcoInf was distantly related to the SPO1-like family of virulent phages, suggesting it is an authentic virulent phage. For bacterial control application purposes, it is important that the genome analysis indicates that both phage are virulent, that is neither phage possesses genes required for lysogenic control. Virulent phages are preferred over temperate phage for bacterial control applications, due to the potential for rapid resistance developing in the bacterial population as a result of prophage immunity to superinfection.

Application of EcoSau and EcoInf was shown to control corn mash contamination by *L. fermentum* strain 0315-25, a strain previously demonstrated to cause aggressive fermentation slowdown [5, 8]. As discussed previously, not all *L. fermentum* isolates are equally effective in inhibiting yeast fermentation and thus *L. fermentum* 0315-25 was chosen to test phage control in corn mash. Inoculation of *L. fermentum* 0315-25 at 10^7^ cfu/ml resulted in a 14 % loss in ethanol yield. This ethanol yield loss is much greater compared to what is typically observed in commercial plants, which is ~2 % loss associated with chronic infection [33]. This bacterial challenge level caused the fermentation to be “stuck” at ~48 h, indicated by weight loss due to CO_2_ evolution (data not shown). The levels of lactic acid and acetic acid in this experiment were also high following bacterial challenge, enough to cause concern during daily operations as 0.2–0.8 % (w/v) lactic acid and 0.05–0.1 % (w/v) acetic acid are believed to be enough to stress yeast [2]. Challenging the systems with 10-fold less *L. fermentum* (10^6^ cfu/ml) resulted in less ethanol loss, and less alarming levels of lactic acid and acetic acid; however, phage treatment still restored fermentation parameters back to the levels found in the unchallenged sample (data not shown). For both levels of bacterial challenge, phage treatments mitigated the effects of *L. fermentum* challenge and restored the levels of ethanol, glucose, lactic acid, and acetic acid to those comparable to the infection-free control. Despite the potential for interference by polysaccharides or solids, here application of phage was shown to restore healthy fermentation characteristics in a corn mash matrix.

## Conclusions

This study clearly demonstrates the predominance of lactic acid bacteria, notably *Lactobacillus* spp., in the bacterial contamination flora of commercial ethanol fermentations. Adding phages EcoSau and EcoInf to *L. fermentum*-contaminated yeast fermentation models resulted in reductions in organic acid levels and restoration of ethanol yields. These fermentation experiments demonstrate that phage efficacy is observed in a corn mash matrix, and support a model in which phage cocktails with broad coverage of major lactic acid bacteria species can be applied directly to corn mash, potentially as antibiotic alternatives in the ethanol fermentation industry.

## Methods

### Bacterial and phage culture conditions

Bacterial strains were routinely cultured at 30 °C using deMan-Rogosa-Sharpe medium (BD Difco, Becton–Dickinson, Sparks, MD, USA) in a hypoxia chamber under controlled gas composition (5.1 % CO_2_, 5.1 % H_2_, 89.8 % N_2_). MRS agar plates contained 1.5 % (w/v) agar (BD Difco). Phage were titered and propagated using the soft agar overlay method [34] on MRS bottom plates and using MRS soft agar [MRS broth plus 0.5 % agar (Bacto) and 20 mM CaCl_2_]. Due to acid production during bacterial growth, the pH of the phage stocks were adjusted to pH 6.5 with NaOH.

### Bacterial diversity analysis of mash samples

Corn mash samples were collected from ethanol plant fermentors at different fermentation stages and cold shipped overnight to our laboratory. For bacterial diversity analysis, total DNA was isolated from each sample using Mo Bio UltraClean™ Microbial DNA Isolation kit (Mo Bio Laboratories, CA, USA). DNA was subject to bacterial tag-encoded FLX amplicon pyrosequencing (bTEFAP) using primers 939F-TTGACGGGGGCCCGCAC and 1492R-TACCTTGTTACGACTT, and resulting sequences were analyzed as previously described [35, 36]. All sequences passing the quality score were compared to a ribosomal database using BLASTn to make taxonomic classifications [37].

### Microbial analysis and bacterial strain isolation from mash samples

Corn mash samples were collected from fermentors of commercial ethanol plants and shipped immediately to the laboratory on ice packs. Analysis of the mash samples were carried out immediately upon sample reception, which is within 20–24 h since sampling from plants. Yeast counts were determined by plating the serial diluted samples onto potato dextrose agar (BD BBL™, Franklin Lakes, NJ, USA) and incubating at 30 °C for 1–2 days. The identity of yeast colonies on the plates was confirmed morphologically by examination under a microscope. Total bacterial counts were determined by serial dilution, plating on MRS agar plates supplemented with 0.05 mg/ml cycloheximide, and incubating in a hypoxic chamber at 30 °C for 1–3 days. From the highest dilution plates, representative bacterial colonies from the predominant colony morphological types were subject to several rounds of sequential colony purification. Resulting isolates were identified via colony PCR for 16S rDNA gene sequencing, using primers 16S.F-CCTACGGGAGGCAGCAG, and 16S.R-CCCCGTCAATTCCTTTGAGTTT. Strains isolated from this study and from previous work [5, 8] were used for phage isolation and characterization.

### Phage isolation and host-range characterization

Phages EcoSau and EcoInf were isolated from commercial sauerkraut and municipal wastewater influent, respectively, using a previously described enrichment method [38]. Briefly, enrichments were set up with 25 ml of filter-sterilized sample liquids and 25 ml double-strength MRS broth to which 1 ml of an overnight *L. fermentum* 0315-25 culture (~OD_600_ = 10) was added. Following 24 h incubation at 30 °C, bacteria were removed by centrifugation (10,000×*g*, 5 min) and passage through 0.22 μm filters. Phage host-range determinations were made using a routine test dilution (RTD) assay, with the RTD defined as the last 10-fold serial dilution that produced confluent clearing when spotted onto a lawn of the phage host. The RTD concentrations determined for EcoSau and EcoInf were both ~10^5^ pfu/ml. Ten microliters of the phages diluted RTD were spotted onto lawns of individual hosts. The spotted plates were incubated at 30 °C for 24–48 h before they were scored for the appearance of clearing, which were interpreted as positive host-range results.

### TEM

Phage lysates were prepared for transmission electron microscopy (TEM) by diluting lysates 1:5 with TEM buffer (20 mM NaCl, 10 mM Tris–HCl pH 7.5, 2 mM MgSO_4_) before applying to 10–15 nm carbon films using the Valentine method [39]. Specimens were stained with 2 % (w/v) aqueous uranyl acetate and observed on a JEOL 1200EX transmission electron microscope operating at an acceleration voltage of 100 kV. Measurements were calibrated using a carbon grating replica (Electron Microscopy Sciences, Hatfield, PA, USA).

### Phage genome sequencing and annotation

Filter-sterilized phage plate lysates were used for phage genomic DNA preparation with the Wizard DNA cleanup kit (Promega, Madison, WI, USA) following the protocols described previously [40]. Phages were sequenced to 23-fold coverage by 454 pyrosequencing (Roche/454 Life Sciences, Branford, CT, USA). Gap closure was completed by amplification of gap regions by PCR followed by Sanger sequencing of the products. The sequences and structure of the genome ends were determined as previously described [40]. Finished DNA sequences were analyzed by Genemark.hmm [41] to detect protein-coding genes. The predicted coding regions were manually edited in Artemis [42]. Predicted proteins were searched against the GenBank nr database using BLASTp [43]. Protein conserved domains were detected with InterProScan version 4.7 run locally [44]. Transmembrane domains and signal sequences were predicted using TMHMM 2.0 (http://www.cbs.dtu.dk/services/TMHMM) and SignalP 3.0 (http://www.cbs.dtu.dk/services/SignalP-3.0/) [45]. Prediction of tRNA genes were carried out using tRNAscan (http://lowelab.ucsc.edu/tRNAscan-SE/) [46]. Phage genome maps were rendered with DNA Master (http://cobamide2.bio.pitt.edu/computer.htm).

### Nucleotide sequence accession number

The genome sequences of phages EcoSau and EcoInf have been deposited in the GenBank database under accession numbers KP027015 (vB_LfeS_EcoSau) and KP054477 (vB_LfeM_EcoInf).

### Ethanol fermentation and phage efficacy testing

Ethanol fermentations were performed in shake flasks as described previously [8]. Corn mash (approximately 33 % solids) was obtained from a commercial dry-grind ethanol facility and stored at −20 °C prior to use. Corn mash (40 ml) was dispensed in 50 ml Erlenmeyer flasks, and supplemented with ammonium sulfate (0.12 % w/v) and glucoamylase (20 μl Optidex L-400, Genencor International Inc., Rochester NY, USA). Each flask was inoculated with *S.cerevisiae* strain NRRL Y-2034 (obtained from the ARS Culture Collection maintained at the USDA-ARS National Center for Agricultural Utilization Research, Peoria, IL, USA) to an initial density of 10^7^ cfu/ml. When indicated, infected samples were inoculated with *L. fermentum* 0315-25 to 10^7^ cfu/ml. Phage-treated samples received either phage EcoInf, phage EcoSau, or the combination of two (1:1 ratio) at a treatment MOI of 10 (10 phage particles per bacterial cell). All groups (infection-free control, infection control, and phage-treated groups) were set up in triplicate and were incubated at 32 °C with shaking (100 rpm) for 72 h. Phage levels in phage-treated groups were determined during fermentation (7, 18, and 24 h). To be specific, 1.5 ml fermentation sample was aseptically taken and centrifuged. The supernatant was filtered through a 0.45 μm filter and the serial dilutions of the filtered sample were plated on the pre-made host lawn. Concentrations of ethanol, glucose, lactic acid, and acetic acid at 72 h were determined by high performance liquid chromatography using a 300 mm Aminex HPX 87H column (Bio-Rad Laboratories, Inc., Hercules, CA, USA) on a HP 1100 Series HPLC system equipped with a refractive index detector (Agilent Technologies, Santa Clara, CA, USA). Samples (10 μl) were injected onto a heated column (65 °C) and eluted at 0.6 ml/min using 5 mM H_2_SO_4_ as mobile phase. Data were reported as the mean value ± standard deviation of triplicate cultures.

## Additional files

Additional file 1:
**Table S1.** Predicted proteins, gene starts and annotations of phage EcoSau. Additional file 2:
**Table S2.** Predicted proteins, gene starts and annotations of phage EcoInf. Additional file 3:
**Table S3.** Relationship of EcoSau to other phages and prophage elements. Additional file 4:
**Table S4.** Relationship of phage EcoInf to SPO1-like phages.

## Abbreviations

LAB: lactic acid bacteria
OUT: operational taxonomic units
cfu: colony forming units
MRS: deMan-Rogosa-Sharpe
TEM: transmission electron microscopy
RTD: routine test dilution
MOI: multiplicity of infection
HPLC: high performance liquid chromatography
BLAST: Basic Local Alignment Search Tool
NCBI: National Center of Biotechnology Information
SRB: sulfate reducing bacteria
