# Supplementary material for: Bacteriophage application restores ethanol fermentation characteristics disrupted by Lactobacillusfermentum
Source: Biotechnol Biofuels. 2015 Sep 4;8:132. doi: 10.1186/s13068-015-0325-9 (PMC4558781; doi:10.1186/s13068-015-0325-9)
Supplement: Additional file 1: — Table S1. Predicted proteins, gene starts and annotations of phage EcoSau. [file 13068_2015_325_MOESM1_ESM.pdf]

Table S1. Predicted proteins, gene starts and annotations of phage EcoSau.

Protein name	Start	Stop	Strand	Upstream Sequence	S-D	Spacing	Start Codon	Predicted product	Evidence	TMHMM
gp01	131	565	+	ttattacagtgcaacgtgaaagggg	None	0	gtg	Terminase small subunit	IPR005335, PF03592	
gp02	549	701	+	aacattgaaat AGGAG tgggagaat	AGGAG	9	atg	Hypothetical novel protein		1
gp03	739	2004	+	gcctttaaggaag GGAGGT gatacc	GGAGGT	6	atg	Terminase large subunit	IPR006701, PF04466	
gp04	2018	3424	+	atctatattta GAGGT ggtgattta	GAGGT	9	atg	phage portal protein	IPR021145, PF05133	
gp05	3399	4430	+	gatatggataaacc GGGT gataata	GGGT	7	atg	phage head morphogenesis protein	IPR006528, PF04233	
gp06	4560	4778	+	ggtttactgttaaag GGGT gataaa	GGGT	6	atg	conserved hypothetical protein		
gp07	4878	5480	+	aaatgcgtgac AGGAGGT ttccaaa	AGGAGGT	7	atg	conserved hypothetical protein		
gp08	5494	6378	+	tttaaccatt AGGAGG aaataaatt	AGGAGG	9	atg	Capsid protein		
gp09	6423	6794	+	tggcgtataaggtt AGGT gattata	AGGT	7	atg	putative DNA packaging	IPR021146, PF05135	
gp10	6784	7092	+	ttcaacccttata GAGGT gatagcc	GAGGT	7	gtg	conserved hypothetical protein		
gp11	7085	7441	+	tattcgctattggt AGGT gaggacg	AGGT	7	ttg	phage head/tail component	IPR006967, PF04883	
gp12	7441	7836	+	atgaattaaag GGGT taatgcaata	GGGT	10	atg	conserved hypothetical protein		
gp13	7852	8451	+	attaatctgaa AGGA ctgtaaataa	AGGA	10	atg	major tail protein	IPR011855, PF06199	
gp14	8530	8862	+	ttttttcgtat GGAGGT aaaaaata	GGAGGT	8	atg	conserved hypothetical protein		
gp15	8530	9302	+	ttttttcgtat GGAGGT aaaaaata	GGAGGT	8	atg	conserved hypothetical protein		
gp16	9295	12414	+	ccttttaaagaa AGGAGGT caggca	AGGAGGT	6	atg	phage tape measure protein	IPR010090, PF10145	3
gp17	12428	13213	+	taccgctttt AGGAGGT gataagac	AGGAGGT	8	gtg	phage tail protein	IPR006520	
gp18	13210	16677	+	acttgacttac GAGGT gaagaaagt	GAGGT	9	atg	phage structural protein	IPR007119	
gp19	16670	16816	+	gtgaaggaact GGAGG ataaattaa	GGAGG	9	atg	Hypothetical novel protein		
gp20	16816	17187	+	aagacaagaag GGAG aagaaaagta	GGAG	10	atg	conserved hypothetical protein		
gp21	17266	17643	+	gcttttaaggaag GGGT gagaagaa	GGGT	8	atg	conserved hypothetical protein		4
gp22	17648	17803	+	ttagtaataaaaag GGGT tgataat	GGGT	7	atg	conserved hypothetical protein		1
gp23	17800	18204	+	aggaccaattgc GGAGG aaaagaaa	GGAGG	8	atg	conserved hypothetical protein		1
gp24	18217	19110	+	agcaaaaggctaa GGGG gaattaaa	GGGG	8	atg	endolysin	IPR002053, IPR010466, PF01183	
gp25	19128	20360	+	ttaaataatagga AGGA aggaacaa	AGGA	8	atg	conserved hypothetical protein		
gp26	20357	20587	+	tcgtagcact AGGAGGT tctttatc	AGGAGGT	8	atg	conserved hypothetical protein		
gp27	20708	20959	+	ttaagaaataga GAGGT aattaatt	GAGGT	8	atg	Hypothetical novel protein		
gp28	21130	21333	+	acaaaggaa AGGAGG acaaaaaata	AGGAGG	10	atg	transcriptional regulator	IPR001387, PF01381	
gp29	21451	21924	+	attaagaaaaga GAGGT aattaatc	GAGGT	8	atg	conserved hypothetical protein	IPR011434, PF07553	1
gp30	21961	22149	+	gaaggaccccgacta GGGT cctttt	GGGT	6	ttg	Hypothetical novel protein		
gp31	22239	22382	+	gttgtggccgtagggg GGAG gacgt	GGAG	5	gtg	Hypothetical novel protein		
gp32	22398	22727	+	aaagtgttgaccttctgaaaaaatc	None	0	gtg	DNA-binding protein	IPR010982	
gp33	22741	22917	+	gacgaagcatagagg GGGT aatcct	GGGT	6	atg	Hypothetical novel protein		1
gp34	22999	23205	+	aagaaccttat AGGAG aattataac	AGGAG	9	atg	conserved hypothetical protein		
gp35	23208	23687	+	acgaagcaacag GGAGGT tttaaga	GGAGGT	7	gtg	conserved hypothetical protein	IPR008840	
gp36	23688	25058	+	taaccgc GGAG tgattattaaataa	GGAG	14	atg	helicase	IPR001650, PF00271	
gp37	25074	25838	+	tatcaaatagagaag AGGAG aacga	AGGAG	5	atg	conserved hypothetical protein		
gp38	25843	26358	+	caaaaagtaaagga AGGT taaaatt	AGGT	7	atg	conserved hypothetical protein		
gp39	26434	27213	+	ctattttttt AGGAG aggtgaaaaa	AGGAG	10	atg	DNA primase	IPR014820, PF08708	
gp40	27214	28446	+	tgatctaagaaac GGGG gacgataa	GGGG	8	atg	conserved hypothetical protein	IPR007936, PF05272	
gp41	28802	29143	+	ggcgggctggtttact GGAG taaca	GGAG	5	atg	DNA nuclease	IPR014883, PF08774	
gp42	29121	29264	+	gttaaaatcgtta AGGA aggattag	AGGA	8	ttg	conserved hypothetical protein		
gp43	29276	29434	+	acaagacgcaat AGGAGG actgtta	AGGAGG	7	atg	conserved hypothetical protein		
gp44	29421	29609	+	aagctattact GGAGG cactgaaga	GGAGG	9	atg	Hypothetical novel protein		
gp45	29634	29930	+	acaaggcacaat AGGAGG ataacta	AGGAGG	7	atg	conserved hypothetical protein		
gp46	29884	30018	+	cggacttatcaagaa GAGG aaagcg	GAGG	6	atg	Hypothetical novel protein		
gp47	30041	30328	+	taaattagaaaggga AGGT acagaa	AGGT	6	atg	conserved hypothetical protein	IPR021739, PF11753	
gp48	30325	30582	+	ccacacat AGGA acagagaggaaaa	AGGA	13	atg	Hypothetical novel protein		
gp49	30685	31119	+	ctgaagataga GAGG aaaaataaca	GAGG	10	atg	conserved hypothetical protein		
gp50	31132	31596	+	gtttgaaaactaaccga AGG gagcg	AGG	5	atg	phage transcriptional regulator	IPR006524	
