# Supplementary material for: Bacteriophage application restores ethanol fermentation characteristics disrupted by Lactobacillusfermentum
Source: Biotechnol Biofuels. 2015 Sep 4;8:132. doi: 10.1186/s13068-015-0325-9 (PMC4558781; doi:10.1186/s13068-015-0325-9)
Supplement: Additional file 2: — Table S2. Predicted proteins, gene starts and annotations of phage EcoInf. [file 13068_2015_325_MOESM2_ESM.pdf]

Table S2. Predicted proteins, gene starts and annotations of phage EcoInf.

Protein name	Start	Stop	Strand	Upstream Sequence	S-D	Spacing	Start Codon	Predicted product	Evidence	TMHMM
gp001	250	678	+	caatcaaaggaa AGGA atattaaac	AGGA	9	atg	hypothetical novel protein		
gp002	1031	2032	-	aggaattaaagaaa AGGAG attatt	AGGAG	6	atg	conserved hypothetical protein	IPR011004	
gp003	2164	2382	-	acaaaagaaa AGGAGG aatataatt	AGGAGG	9	atg	hypothetical novel protein		
gp004	2515	2763	-	gacaaatgaag GGAG tgaatagcca	GGAG	10	atg	hypothetical novel protein		
gp005	2780	3952	-	atataaattagta AGGT gaaatatc	AGGT	8	ttg	conserved hypothetical protein		
gp006	4064	4837	-	gtggata AGG cactatatagatata	AGG	15	gtg	Nicotinamide mononucleotide transporter PnuC	IPR006419, PF04973	8
gp007	4910	5059	-	tagaaaaggaaaggg GGA caaaaac	GGA	7	atg	conserved hypothetical protein		
gp008	5179	8691	+	taaatgtaatta AGGT ggtggaact	AGGT	9	atg	putative tape measure-like protein	IPR002901, PF01832	
gp009	8803	9240	-	ctaaacttttt AGGAGGT tttaata	AGGAGGT	7	ttg	tail tube subunit		
gp010	9337	9591	-	acttgtttaaggg GGA acattaaaa	GGA	9	atg	hypothetical novel protein		1
gp011	9594	9884	-	ggaatgattgtagg GGGT aaagata	GGGT	7	atg	conserved hypothetical protein		
gp012	9890	10225	-	gggacggtttagg GGGT tattaaat	GGGT	8	atg	conserved hypothetical protein		
gp013	10228	10557	-	tgtgatgattaata GGGG gagaact	GGGG	7	atg	hypothetical novel protein		
gp014	10571	10852	-	ttgaattttaca GAGGT gattaagt	GAGGT	8	atg	hypothetical novel protein		
gp015	11059	11313	-	aattaggaaaggg GGAG ttaattaa	GGAG	8	atg	hypothetical novel protein		2
gp016	11438	12406	+	attaaactcgc AGGAG atgattaat	AGGAG	9	gtg	Mg2+ transporter protein	IPR002523, PF01544	2
gp017	12452	12637	-	aaaaaagaaaagga AGGT aataata	AGGT	7	atg	hypothetical novel protein		1
gp018	12769	14796	-	gcgacaatagtta GGGG gcatagtt	GGGG	8	atg	conserved hypothetical protein		
gp019	14808	15809	-	gattaagtaaga AGGT ggtgattaa	AGGT	9	atg	conserved hypothetical protein		
gp020	15829	16077	-	accaacagga AGGA tttgacatttc	AGGA	11	atg	hypothetical novel protein		
gp021	16074	17903	-	acctaatgaaagcaag GGA aagtga	GGA	6	ttg	DNA helicase	IPR001650, PF00271	
gp022	17900	19012	-	taccatagttttgg GGGT gaagccg	GGGT	7	atg	conserved hypothetical protein	IPR004843, PF00149	
gp023	19030	19416	-	taaaaattgaaaga AGGA ctgtaaa	AGGA	7	atg	hypothetical novel protein		
gp024	19462	19752	-	gaaaactaaataa AGGAG aagaata	AGGAG	7	gtg	conserved hypothetical protein		
gp025	19752	19952	-	gcagagacaactaa AGGAG ataaga	AGGAG	6	atg	hypothetical novel protein		
gp026	19939	20244	-	gaaaggtaactaa AGGAG ataaaaa	AGGAG	7	atg	conserved hypothetical protein		
gp027	20241	20462	-	agtaggggtttatgg GGGT gatcaa	GGGT	6	atg	conserved hypothetical protein		
gp028	20492	20788	-	tctataatgtt GAGGT ggataaata	GAGGT	9	atg	conserved hypothetical protein		
gp029	20788	21006	-	aattatttgtt AGGAG tgatgatag	AGGAG	9	atg	hypothetical novel protein		
gp030	21231	21386	-	caaaaagaaaagga AGGT actaata	AGGT	7	atg	hypothetical novel protein		
gp031	21812	23680	-	ataacttaacagaaaa GGGG attat	GGGG	5	atg	tail sheath protein	IPR007067, PF04984	
gp032	23761	24693	-	tttattgtatattaagtaaagatag	None	0	gtg	conserved hypothetical protein		
gp033	25310	26059	-	ggaataaagaaaga AGGA aacaaac	AGGA	7	atg	hypothetical novel protein		
gp034	26233	26775	-	aaaaaaatgaaaga AGGA attcaaa	AGGA	7	gtg	DNA helicase	IPR007694, PF03796	
gp035	26925	27149	-	aaaaatagtttggt AGGT gatgata	AGGT	7	atg	hypothetical novel protein		
gp036	27139	30114	-	gaaaagaaagt AGGA agataacaag	AGGA	10	atg	DNA polymerase	IPR001098, PF00476	
gp037	30127	30264	-	ttaagtttaagg GGGT ggtaaaaac	GGGT	9	atg	hypothetical novel protein		1
gp038	30899	31420	-	gctataacaagc AGGT gaagaacat	AGGT	9	atg	conserved hypothetical protein		
gp039	31439	31768	-	ggaaccatgaaatagaaa AGG agca	AGG	4	atg	hypothetical novel protein		
gp040	32523	32675	-	tgattaatattg GGA aacaccgagt	GGA	10	ttg	hypothetical novel protein		
gp041	33262	33411	+	aaaatctattgacaatt AGG acgga	AGG	5	gtg	hypothetical novel protein		1
gp042	33433	33603	+	ttaataagaatgtc AGGT tattaag	AGGT	7	atg	hypothetical novel protein		
gp043	33591	33833	+	tgcatgaagtgg GGGT agaagaact	GGGT	9	atg	hypothetical novel protein		
gp044	33850	34299	+	cacgagtaatta AGGAGGT ttcatc	AGGAGGT	6	atg	hypothetical novel protein	IPR014905, PF08797	
gp045	34452	35024	+	aaagttggtatt AGGT gttgggtta	AGGT	9	atg	Peptidoglycan-binding protein LysM	IPR018392, PF01476	
gp046	35102	35260	+	gggtataaagaa AGGAG ttgaaata	AGGAG	8	atg	conserved hypothetical protein		
gp047	35283	35723	+	tagaatcaagaa AGGA atgatacgt	AGGA	9	atg	hypothetical novel protein		
gp048	36119	36385	+	attcttt GGT ggcccttggggatcc	GGT	15	atg	hypothetical novel protein		3
gp049	36378	36767	+	agttctgatgataat GGAGG taata	GGAGG	5	atg	hypothetical novel protein		1
gp050	36754	37197	+	gatcaggcaa AGGAG attaatcaaa	AGGAG	10	atg	Holin		1
gp051	37286	38755	+	aaagaagaaagg GGA cttaattttc	GGA	10	atg	Lysin	IPR002053, PF01476	1
gp052	38907	39578	+	acaagtacagaaaag GGGT ttataa	GGGT	6	atg	conserved hypothetical protein		
gp053	39599	39970	+	attaattaagtaa GGGG gcaaaact	GGGG	8	ttg	hypothetical novel protein		
gp054	39973	40524	+	cagacccacagga AGGT gaataatt	AGGT	8	ttg	hypothetical novel protein		
gp055	40689	40856	+	tattcacactctaa GGGT ttgtgca	GGGT	7	ttg	conserved hypothetical protein		
gp056	40856	41269	+	aaaggaagg GGGT tggcaatagcta	GGGT	12	atg	hypothetical novel protein		
gp057	41270	43111	+	taatttaaaagcaaacgaagggtag	None	0	gtg	Terminase	IPR008866, PF05876	
gp058	43108	44658	+	tgacagatgtaa AGGA atttggtga	AGGA	9	gtg	Phage portal protein	IPR006944, PF04860	
gp059	44909	46399	+	agatgaa AGGA agttattagaacat	AGGA	14	atg	major capsid		
gp060	46507	47367	+	tatagtatgtattggt GGT gatttg	GGT	6	gtg	Prohead peptidase	IPR006433, PF04586	
gp061	47385	48314	+	atgattaa AGGAG atttataaagat	AGGAG	12	atg	hypothetical novel protein		
gp062	48421	48651	-	agatattaaaaca AGGT ggtaaaac	AGGT	8	atg	hypothetical novel protein		
gp063	48655	49491	-	agtacaaccaaa AGGAGG ctatgta	AGGAGG	7	atg	conserved hypothetical protein		
gp064	49491	50204	-	tgaatgacttctt AGGT gattaatc	AGGT	8	atg	conserved hypothetical protein		
gp065	50207	51181	-	ttaaataagaaatggt GGT gattaa	GGT	6	atg	conserved hypothetical protein		
gp066	51297	51512	+	taagataataaa AGGAG tgattaat	AGGAG	8	ttg	hypothetical novel protein		
gp067	51520	51777	+	ttagaagaag GGA agtagtaatact	GGA	12	atg	hypothetical novel protein		
gp068	51778	52422	+	caagaaacaac GAGGT gaagattag	GAGGT	9	atg	conserved hypothetical protein		
gp069	51778	52966	+	caagaaacaac GAGGT gaagattag	GAGGT	9	atg	conserved hypothetical protein		
gp070	53023	56259	+	attacgattgat AGGT ggtgaaata	AGGT	9	atg	Tape measure protein	See text	
gp071	56272	56616	+	ctactcacatta AGGAGG aactaga	AGGAGG	7	atg	conserved hypothetical protein		
gp072	56661	61163	+	aaccttatagaa AGGA ctaaaatta	AGGA	9	atg	conserved hypothetical protein		
gp073	61233	61823	+	atttgaataagggg GGA taaaataa	GGA	8	atg	conserved hypothetical protein		
gp074	61832	62401	+	taaatgatgggaagt AGGT gagtag	AGGT	6	atg	conserved hypothetical protein		
gp075	62411	63124	+	tgggtatacaaaata GGGG ggtggc	GGGG	6	gtg	GPW/gp25 family protein	IPR007048, PF04965	
gp076	63136	64692	+	tctcactaagat AGGAGG gaactaa	AGGAGG	7	atg	Baseplate J family protein	IPR006949, PF04865	
gp077	64693	66591	+	tagtgttgttgacg GGGT tgattaa	GGGT	7	atg	conserved hypothetical protein		
gp078	66603	67142	+	tggaaacaaattaagc GAGG taaat	GAGG	5	atg	conserved hypothetical protein		
gp079	67157	69748	+	tcaagttttaggaa GGGT ggtgtat	GGGT	7	atg	conserved hypothetical protein		
gp080	69764	70885	+	acttaattaac AGGAGG aataaagt	AGGAGG	8	atg	conserved hypothetical protein	IPR007110, PS50835	
gp081	70885	71058	+	aatatacttgc GAGGT aaatcaata	GAGGT	9	atg	conserved hypothetical protein		
gp082	71039	77122	+	gaaatttgggc GGAGG aaactgaca	GGAGG	9	atg	conserved hypothetical protein		
gp083	77149	77391	+	catgcaatataagtag GGAG gaaac	GGAG	5	atg	hypothetical novel protein		
gp084	77401	77634	+	tatgtcaagtaaata AGGAG gtact	AGGAG	5	atg	hypothetical novel protein		
gp085	77715	78440	+	actatcactaagga AGGT gataatc	AGGT	7	atg	hypothetical novel protein		
gp086	78422	79888	+	atcaattagcaa AGGA aaatcttca	AGGA	9	atg	DNA helicase	IPR007694, PF03796	
gp087	79892	82885	+	taatgcaatcaagg GGGT atagtcc	GGGT	7	ttg	endonuclease	IPR004042, PF05204	
gp088	82900	83514	+	tagaactgta AGGAGG attatttaa	AGGAGG	9	atg	conserved hypothetical protein		
gp089	83507	84556	+	aagatagtta AGGT acgtgatcgtt	AGGT	11	atg	DNA primase	IPR002694, PF01807	
gp090	84558	84911	+	ctaaatatgtta GGGG gacgataga	GGGG	9	atg	hypothetical novel protein		
gp091	84904	85224	+	caattaaaaagtg AGGA caacaata	AGGA	8	atg	hypothetical novel protein		
gp092	85227	85925	+	aaggggatcaa GGGG ataagtaata	GGGG	10	atg	hypothetical novel protein		
gp093	86005	86652	+	atttaaactatgaa AGGA aaaagaa	AGGA	7	atg	conserved hypothetical protein		
gp094	86655	87263	+	atttagttgataatattaaataact	None	0	atg	conserved hypothetical protein		
gp095	87335	87802	+	catcgaaaggaa AGGAG atctagtc	AGGAG	8	atg	conserved hypothetical protein		
gp096	87802	88437	+	gggcagaacgga AGGT ggaacacta	AGGT	9	atg	conserved hypothetical protein	IPR011630, PF07659	
gp097	88479	89657	+	acatattaaa AGGAGG aattaaatc	AGGAGG	9	atg	conserved hypothetical protein		
gp098	89709	90236	+	ggggctttttttatta GGGG atcaaa	GGGG	6	atg	conserved hypothetical protein	IPR011004	
gp099	90267	90473	+	ggttttttat AGGAG aatcatagttt	AGGAG	11	atg	conserved hypothetical protein		

gp100	90533	91150	+	tatgattaaagaaa AGGAG attctt	AGGAG	6	atg	conserved hypothetical protein	IPR011004	
gp101	91169	91624	+	tgactaacagttaggg GGGT atcaa	GGGT	5	atg	conserved hypothetical protein	IPR011004	
gp102	91643	92902	+	ttgctaatagct AGGAGGT taaaaa	AGGAGGT	6	atg	DNA recombinase RecA	IPR013765, PF00154	
gp103	92892	93254	+	gctaaggaagt GGGG gaaattctca	GGGG	10	atg	hypothetical novel protein		
gp104	93329	94024	+	ctttttgtgttaaag GGGT aaatt	GGGT	5	atg	conserved hypothetical protein		
gp105	94106	94489	+	aaaggataaaagaa AGGAG tttctt	AGGAG	6	atg	conserved hypothetical protein	IPR011004	
gp106	94510	94866	+	tctaatggttgg GGGT atttaaaga	GGGT	9	atg	hypothetical novel protein	IPR019096, PF09643	
gp107	94886	95158	+	aaatagaaaggg GGA aataatttaa	GGA	10	atg	hypothetical novel protein		
gp108	95250	96743	+	ttcttagataaatgc GAGGT ttatt	GAGGT	5	atg	conserved hypothetical protein		
gp109	96754	97254	+	ttcaccttcttgta AGGAG ctgtat	AGGAG	6	atg	hypothetical novel protein		2
gp110	97244	97792	+	ttaaacctaactag GGGT gatcaaa	GGGT	7	gtg	conserved hypothetical protein		
gp111	97785	98432	+	tacatggactatttc AGGT atggga	AGGT	6	atg	conserved hypothetical protein		
gp112	98429	98980	+	tccatgatacaacg GGGT atcaggt	GGGT	7	atg	conserved hypothetical protein		
gp113	98981	99334	+	acgtcgaaaatata AGGAG tattaa	AGGAG	6	atg	hypothetical novel protein		
gp114	99593	100303	+	taaaatagcctag GGGT gaaataag	GGGT	8	atg	conserved hypothetical protein		
gp115	100311	100904	+	ggccta GAG atgaattaaaggagta	GAG	16	atg	hypothetical novel protein		
gp116	100909	101682	+	gcttattacgaag GGA agtaagatc	GGA	9	atg	hypothetical novel protein		
gp117	102007	102192	+	cgtagaaacgaagg GGGT tatagat	GGGT	7	atg	hypothetical novel protein		1
gp118	102176	102331	+	ggggcgtttgtgct AGGT tccttat	AGGT	7	atg	hypothetical novel protein		
gp119	102438	102881	+	ttgaaccaacttgcagaattagact	None	0	atg	conserved hypothetical protein		
gp120	102901	103383	+	gactaaaattta GGGG gatataaga	GGGG	9	ttg	hypothetical novel protein		
gp121	103460	103993	+	gaattaaagaaaag AGGA aattaaa	AGGA	7	atg	conserved hypothetical protein		
gp122	104085	104453	+	gataaacaaaaa GGGG ctaatcaaa	GGGG	9	atg	conserved hypothetical protein		
gp123	104605	105105	+	GGGT gttcactaccagaatggaaac	GGGT	21	atg	DNA polymerase III, beta chain	IPR001001	
gp124	105504	105932	+	caatcaaaggaa AGGA atattaaac	AGGA	9	atg	hypothetical novel protein		
