# Supplementary material for: Bacteriophage application restores ethanol fermentation characteristics disrupted by Lactobacillusfermentum
Source: Biotechnol Biofuels. 2015 Sep 4;8:132. doi: 10.1186/s13068-015-0325-9 (PMC4558781; doi:10.1186/s13068-015-0325-9)
Supplement: Additional file 3: — Table S3. Relationship of EcoSau to other phages and prophage elements. [file 13068_2015_325_MOESM3_ESM.pdf]

Table S3. Relationship of EcoSau to other phages and prophage elements.

Phage or prophage (host)	Accession No.	Phage/prophage genome length (bp)	Total ORFs	No. of shared proteins with Sau[a]	Identify range of shareded proteins[b]
TP-778L (*Streptococcus thermophilus*)	NC_022776	41757	52	15	2% - 50%
NF5 (*Brochothrix thermosphacta*)	NC_015252	36953	58	13	11% - 38%
TP901-1 (*Lactococcus lactis*)	NC_002747	37667	56	12	11% - 49%
Tuc2009 (Lactococcus lactis)	NC_002703	38347	56	12	10% - 49%
ul36 (*Lactococcus lactis*)	NC_004066	36798	59	10	13% - 41%
P335 (Lactococcus lactis)	DQ_838728	33613	56	10	13% - 43%
2972 (*Streptococcus thermophilus*)	NC_007019	34704	44	9	24% - 52%
Sfi11 (*Streptococcus thermophilus*)	NC_002214	39807	53	9	22% - 51%
phiEf11 (*Enterococcus faecalis*)	NC_013696	42822	65	9	13% - 41%
Prophage Lj928 (*Lactobacillus johnsonii*)	NC_005354	38384	48	8	30% - 72%

Bacterial genome with prophage elements	Accession No.	Prophage element length (bp) / locus tag range	Total ORFs	No. of shared proteins with Sau[a]	Identify range of shareded proteins[b]
Lactobacillus johnsonii FI9785	NC_013504	35660 (FI9785_801 to FI9785_849)	49	25	13% - 73%
Lactobacillus acidophilus 30SC	NC_015214	36334 (LAC30SC_06450 to LAC30SC_06735)	58	15	23% - 72%
Pediococcus pentosaceus ATCC 25745	NC_008525	38079 (PEPE_0745 to PEPE_0807)	63	12	15% - 60%
Lactococcus lactis subsp. *lactis* CV56	NC_017486	50457 (CVCAS_1829 to CVCAS_1886)	58	12	11% - 50%
Leuconostoc sp. C2	NC_015734	37554 (LGMK_06850 to LGMK_07110)	53	12	13% - 61%
Lactobacillus casei BL23	NC_010999	40752 (LCABL_12880 to LCABL_13480)	61	11	6% - 58%
Lactococcus lactis subsp. *cremoris* SK11[c]	NC_008527	65114 (LACR_1080 to LACR_1158)	79	11	13% - 37%
Lactobacillus rhamnosus GG	NC_013198	40161 (LGG_01086 to LGG_01143)	58	11	6% - 57%
Streptococcus pyogenes Alab49	NC_017596	65724 (SPYALAB49_000980 to SPYALAB49_001059)	80	10	17% - 42%
Lactobacillus plantarum WCFS1	NC_004567	60286 (lp_2394 to lp_2477)	85	9	6% - 57%
Streptococcus equi subsp. *equi* 4047	NC_012471	41823 (SEQ_1726 to SEQ_1764)	39	8	15% - 58%

[a]Based on e value $\leq 10^{-5}$

[b]Protein identity is based on Dice value.

[c]There are two prophage elements shared protein identity with Sau, only one region is shown.
