# Supplementary material for: Bacteriophage application restores ethanol fermentation characteristics disrupted by Lactobacillusfermentum
Source: Biotechnol Biofuels. 2015 Sep 4;8:132. doi: 10.1186/s13068-015-0325-9 (PMC4558781; doi:10.1186/s13068-015-0325-9)
Supplement: Additional file 4: — Table S4. Relationship of phage EcoInf to SPO1-like phages. [file 13068_2015_325_MOESM4_ESM.pdf]

Table S4. Relationship of EcoInf to SPO1-like phages.

Phage	Genome accession number	Genome size (kb)	Terminal redundency (kb)	No. of total proteins in the genome	No. of shared proteins with Inf (identity > 25%) [a]	Identity range of shared proteins[a]	No. of shared proteins with Inf (e value < 10^{-5})
Bacillus phage phiAGATE	NC_020081	149.844	2.669	204	12	22% -47%	39
Bacillus phage SPO1	NC_011421	145.747	13.185	204	12	26%-44%	21
Brochothrix phage A9	NC_015253	appx. 127[b]	not known	198	15	26%-47%	30
Enterococcus phage phiEF24C	NC_009904	142.072	circularly permuted[c]	221	17	26%-53%	35
Lactobacillus phage Lb338-1	NC_012530	141.832	nonredundent[c]	199	20	25%-88%	32
Lactobacillus phage LP65	NC_006565	131.573	nonredundent[c]	165	29	27%-62%	41
Listeria phage A511	NC_009811	137.619	3.125	199	22	25%-53%	35
Staphylococcus phage K	NC_005880	127.395[b]	appx. 20	118	19	25%-50%	32
Staphylococcus phage Twort	NC_007021	130.706	not determined	195	19	26%-48%	32

NA, Not available

[a]Protein identities were calculated by the Dice method using BlastP alignment identity scores.

[b]genome size without terminal redundency

[c]as reported by authors
